# Single Nucleotide Polymorphism in *KIR2DL1* Is Associated With HLA-C Expression in Global Populations

**DOI:** 10.3389/fimmu.2020.01881

**Published:** 2020-08-21

**Authors:** Luciana de Brito Vargas, Renata M. Dourado, Leonardo M. Amorim, Brenda Ho, Verónica Calonga-Solís, Hellen C. Issler, Wesley M. Marin, Marcia H. Beltrame, Maria Luiza Petzl-Erler, Jill A. Hollenbach, Danillo G. Augusto

**Affiliations:** ^1^Programa de Pós-Graduação em Genética, Departamento de Genética, Universidade Federal do Paraná, Curitiba, Brazil; ^2^Department of Neurology, Weill Institute for Neurosciences, University of California, San Francisco, San Francisco, CA, United States

**Keywords:** NK cells, KIR, natural selection, linkage disequilibrium, coevolution, expression, population genetics

## Abstract

Regulation of NK cell activity is mediated through killer-cell immunoglobulin-like receptors (KIR) ability to recognize human leukocyte antigen (HLA) class I molecules as ligands. Interaction of KIR and HLA is implicated in viral infections, autoimmunity, and reproduction and there is growing evidence of the coevolution of these two independently segregating gene families. By leveraging *KIR* and *HLA-C* data from 1000 Genomes consortium we observed that the *KIR2DL1* variant rs2304224^*^T is associated with lower expression of HLA-C in individuals carrying the ligand HLA-C2 (*p* = 0.0059). Using flow cytometry, we demonstrated that this variant is also associated with higher expression of KIR2DL1 on the NK cell surface (*p* = 0.0002). Next, we applied next generation sequencing to analyze *KIR2DL1* sequence variation in 109 Euro and 75 Japanese descendants. Analyzing the extended haplotype homozygosity, we show signals of positive selection for rs4806553^*^G and rs687000^*^G, which are in linkage disequilibrium with rs2304224^*^T. Our results suggest that lower expression of HLA-C2 ligands might be compensated for higher expression of the receptor KIR2DL1 and bring new insights into the coevolution of *KIR* and *HLA*.

## Introduction

The *killer cell immunoglobulin-like receptor* (KIR) genes on chromosome 19 encode receptors that interact with a subset of human leukocyte antigen (HLA) class I molecules, encoded by genes on chromosome 6, to regulate NK cell cytotoxicity against infected and neoplastic cells (1–3). In fact, combinations of variants of *KIR* and *HLA* have been repeatedly associated with autoimmune disease (4–6), cancer (7, 8), viral infections (9, 10), and are also implicated in reproduction (11–14). As a result, the interaction of KIR and HLA is relevant to fitness and survival and candidate for evolutionary studies (15).

KIR recognize subsets of HLA-A (A3, A11, and Bw4), HLA-B (Bw4 and Bw6), and HLA-C (C1 and C2) molecules (16). *HLA-C* appears to have had a great impact on *KIR* evolution, driving the expansion of lineage III KIR, which are the receptor lineage that recognize HLA-C (17, 18). The dimorphism in position 80 of HLA-C defines HLA-C1 (80^Asn^) and HLA-C2 (80^Lys^) and confers differential specificity to KIR. Among all ligands, the interaction between KIR2DL1 and HLA-C2 is responsible for the strongest regulatory signal and HLA-C seems to act as the main educator of NK cells (19, 20).

Worldwide studies demonstrate coordinated frequencies of *KIR* and *HLA* in populations. In a comprehensive study consisting of 30 populations, Single et al. (21) found that increasing frequencies of activating KIR are correlated with decreased frequencies of their respective HLA ligands. On the other hand, Hollenbach et al. (22) showed positive correlation between the presence of KIR2DL3 and the presence of HLA-C1 in 105 worldwide populations. A strong and negative correlation of *KIR* gene-content haplotype *A* and HLA-C2, a pair which is associated with increased risk of pre-eclampsia, was found in eight populations from European, African, and Asian ancestries (11). Moreover, there is extensive evidence of balancing selection maintaining diversity in *KIR* genes (23–25). *KIR* and *HLA* segregate independently and there are no reports of gametic association between these two gene families. Here, we show that a single nucleotide polymorphism (SNP) in *KIR2DL1* is associated with expression levels of the KIR2DL1 receptor on the cell surface and also with HLA-C expression.

## Results

### *KIR2DL1* Variant rs2304224^*^T Is Associated With Lower Expression Levels of HLA-C

To search for possible signals of coevolution between *KIR* and *HLA*, we evaluated if variants in inhibitory KIR that bind to HLA-C could be associated with HLA-C expression levels in global populations. We leveraged the public sequencing information available for all populations in the 1000 Genomes Project (1KGP) (26) and retrieved the genotypic data available for SNPs located within *KIR2DL1* and *KIR2DL23* (*rs2304224, rs11673144, rs12982263, rs34721508, rs35719984*, and *rs35861855*) in 955 individuals of various ancestries. We also obtained *HLA* genotyping data available for those individuals (27).

**Figure 1:**
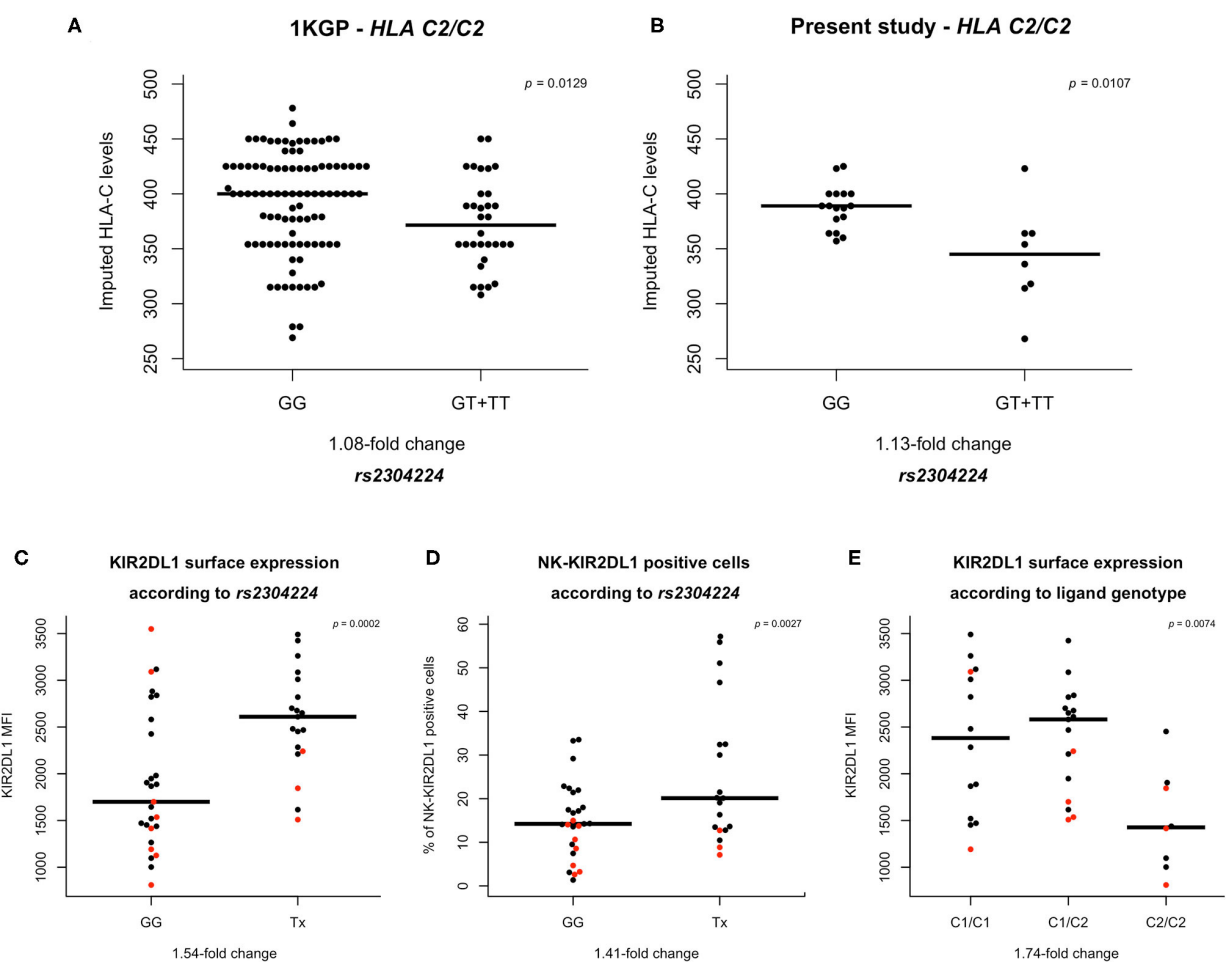
HLA-C and KIR2DL1 expression are associated with genetic variants. **(A,B)** *rs2304224* in *KIR2DL1* marks *in silico* HLA-C surface expression (28) in two different cohorts. The presence of allele rs2304224*T marks lower HLA-C expression in **(A)** 130 *C2/C2* homozygotes out of 955 individuals from 1000 genomes consortium and **(B)** 25 *C2/C2* homozygotes out of 308 Euro-Brazilians from Curitiba (present study). **(C)** Higher KIR2DL1 surface expression and **(D)** increased presence on NK cells are also associated with the variant rs2304224*T (*p* = 0.0002 and *p* = 0.0027, respectively). **(E)** *HLA-C* genotype is associated to KIR2DL1 surface expression (*p* = 0.0074). There is no difference in expression, however, between homozygotes *C1/C1* and heterozygotes *C1/C2* (*p* = 0.44). Homozygosity for *C2/C2*, on the other hand, is associated with lower KIR2DL1 surface expression than in *C1/C1* (*p* = 0.0031) and *C1/C2* (*p* = 0.0016). Each dot in the graphs represents one individual. Red dots indicate hemizygosity for *KIR2DL1*. Median values are shown in horizontal lines and statistical significance is indicated in the top right corners of each plot.

Subsequently, we used previously published data of HLA-C expression levels (28) and imputed the expression for each *HLA-C* genotype in the 1KGP cohort. The variant rs2304224^*^T was associated with lower HLA-C expression levels in individuals *HLA-C1/C2* (*p* = 0.0420) and *HLA-C2/C2* (*p* = 0.0059), but not in *HLA-C1/C1* individuals (*p* = 0.0740; Figure 1A and Supplementary Figure 1). This variant is in position 13 of exon 1 and causes a phenylalanine to valine change in the KIR2DL1 signal peptide. We replicated these results by imputing the HLA expression in an independent panel of 308 Brazilians Euro-descendants for which *HLA* genotyping data was available, and we sequenced the first exon of *KIR2DL1* to genotype *rs2304224* (*p* = 0.0107; Figure 1B).

To demonstrate that our approach to impute the HLA-C expression is predictive of the cell surface expression *in vivo*, we measured the HLA-C surface levels of fresh CD3^+^ cells in 30 individuals using flow cytometry and compared to the imputed values. We found a correlation of *r* = 0.62, *p* < 0.0001 (Supplementary Figure 2).

### rs2304224^*^T Is also Associated With Higher Surface Expression Levels of *KIR2DL1*

We sought to investigate if the variant rs2304224^*^T in *KIR2DL1* was associated with KIR2DL1 surface expression. We used flow cytometry to quantify both the abundance of KIR2DL1 on the surface of NK cells (median fluorescence intensity, MFI) as well as the percentage of NK cells expressing KIR2DL1 on their surface (KIR2DL1^+^ NK cell), and also interrogated if copy number variation of *KIR2DL1* affects surface expression. Although borderline, we did not find significant differences of expression levels in individuals carrying one copy (hemizygous) or two copies (homo- or heterozygous) of *KIR2DL1*^+^ (*p* = 0.0594; Supplementary Figure 3A). However, the number of KIR2DL1+ NK cells was 2.16-fold higher in individuals carrying two copies (*p* = 0.0001; Supplementary Figure 3B). For all KIR2DL1 expression analyses, we used copy number of *KIR2DL1* as covariant in the regression model.

We observed that the allele rs2304224^*^T, associated with decreased HLA-C expression, was also associated with 1.54-fold increase of the KIR2DL1 surface expression (*p* = 0.0002) and a 1.41-fold increase of KIR2DL1^+^ NK cells (*p* = 0.03; Figures 1C,D). The median expression of each KIR allotype is shown in Supplementary Figure 4. We also observed that KIR2DL1 expression was decreased in individuals homozygous for the presence of the C2 ligand (*C2/C2, p* = 0.007; Figure 1E).

### Signals of Positive Selection for *KIR2DL1* Variants in Linkage Disequilibrium With *rs2304224*

We next analyzed the entire *KIR2DL1* gene in a subset of 109 Euro-descendants and 75 Japanese descendants sequenced using our custom next generation sequencing method (29). In Euro descendants, we observed low correlation but strong linkage disequilibrium (LD) between *rs2304224* and three other variants (Supplementary Figures 5A,B). The first variant is at position −406 upstream of the *KIR2DL1* gene (*rs4806553*, D′ = 0.99, *r*^2^ = 0.18, *p* < 10^−8^). The other variants are located within the coding region, in exon 4 (*rs687000*, D′ = 0.99, *r*^2^ = 0.52, *p* < 10^−12^) and exon 7 (*rs34721508*, D′ = 0.99, *r*^2^ = 0.24, *p* < 10^−3^). Weaker LD was observed for the same variants in Japanese descendants (Supplementary Figures 5C,D). Frequencies for all SNPs in both populations are given in Supplementary Table 1. Moreover, the frequency of HLA-C2 in our Japanese-descendant cohort was 10.3% while in Euro-descendants it was 40.9%.

**Figure 2:**
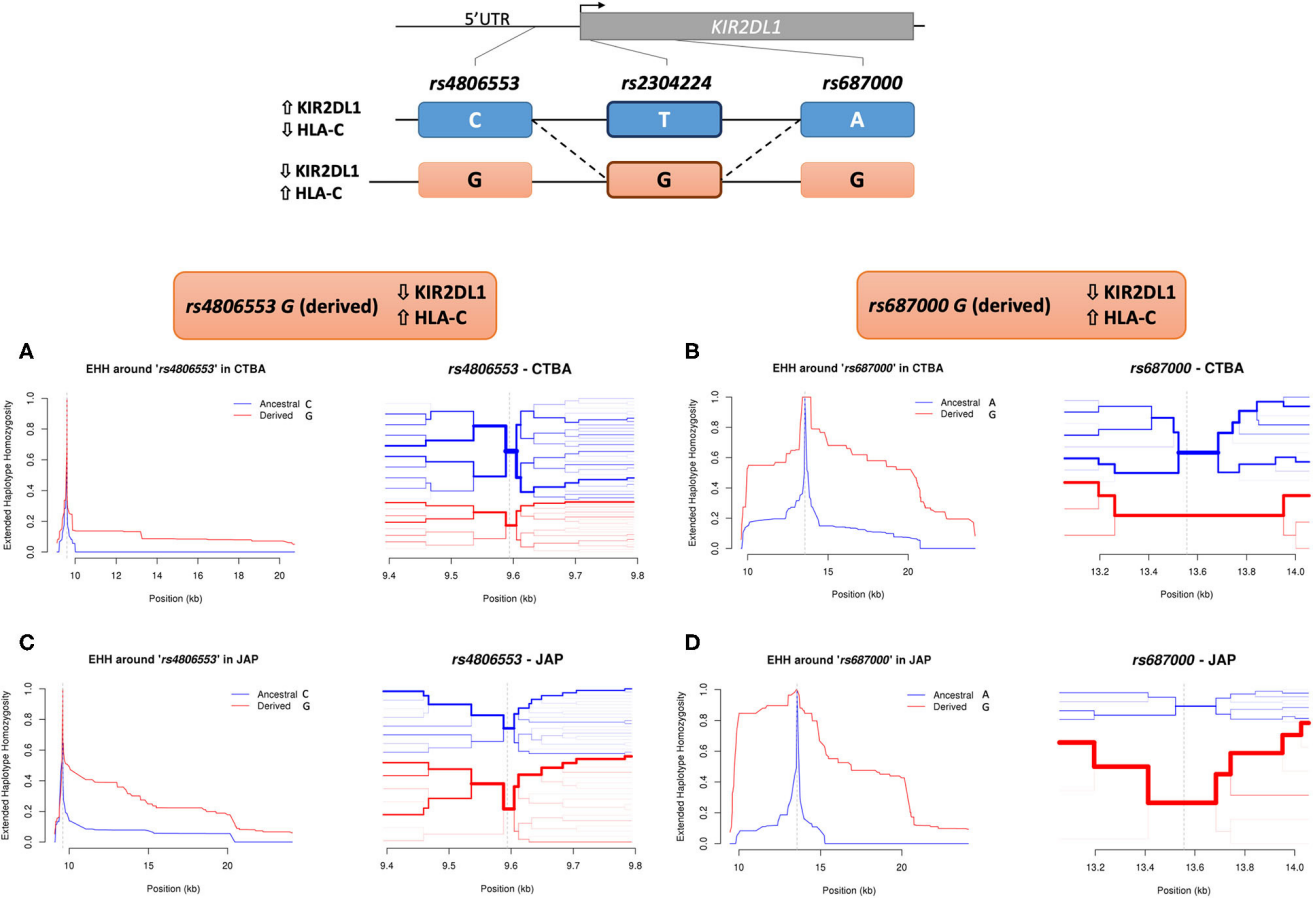
Extended haplotype homozygosity (EHH) in *KIR2DL1*. The extended homozygosity analysis is based on the premise that advantageous alleles increase in frequency at a higher pace than the local recombination rate breaks down the haplotypes in which these alleles are located. Therefore, alleles marking regions with elevated extended homozygosity are possibly under recent positive selection. Here we identify extended haplotypes surrounding *KIR2DL1* variants *rs4806553* and *rs687000*. The possible haplotypes of *rs4806553* and *rs687000* in relation to *rs2304224* are represented at the top of the image. The continuous line represents the most common configuration between two variants, and the dashed line represent less frequent configurations. On the left of each haplotype, arrows indicate higher or lower expression of KIR2DL1 and HLA-C, as associated with *rs2304224* alleles *G* or *T*. A representation of the genomic organization of *KIR2DL1* with the indicated location of the three variants is represented above. **(A)** EHH graph of decay in homozygosity (left) and furcation plot (right) for *rs4806553* in Euro-Brazilians. The graph shows little to no difference between ancestral rs4806553*C (blue) and derived rs4806553*G (red) alleles in Euro-Brazilians. **(C)** EHH graph of decay in homozygosity (left) and furcation plot (right) for *rs4806553* in Japanese. In Japanese, elevated homozygosity is associated with derived allele rs4806553*G (red). **(B)** EHH graph of decay in homozygosity (left) and furcation plot (right) for *rs687000* in Brazilians with European ancestry and **(D)** Brazilians with Japanese ancestry. Elevated homozygosity associated with derived allele rs687000*G (red) is consistent with the selective sweep model, in which recent positive selection sweeps the diversity on nearby loci. Vertical dotted lines indicate the position of the core SNP. The thickness of each branch in the furcation plot is determined by haplotype frequency.

We searched for signals of population specific selection, for both Euro and Japanese descendants, by estimating the extended haplotype homozygosity (EHH) using *rs2304224* and also variants in significant LD with it as focal SNPs. The bifurcation patterns are consistent with positive selection increasing frequencies of the haplotype more rapidly than they could be broken by genetic recombination. Signals of positive selection were observed for the derived allele rs4806553^*^G in Japanese but not in Euro-descendants (Figures 2A,C). Strong signals of positive selection were also observed for the derived allele rs687000^*^G in both Euro and Japanese descendants (Figures 2B,D).

## Discussion

Previous results show that *cis* polymorphisms associated with *HLA-C* expression do not associate with NK cell activity (30), despite the compelling evidence that KIR-HLA are coevolving as an integrated system (11, 16, 21, 22). Here, we show evidence of coevolution of *KIR* and *HLA* by identifying a variant in *KIR2DL1* that was associated with surface expression of the ligand HLA-C2 in worldwide populations. The allele rs2304224^*^T was associated with lower expression of imputed HLA-C surface expression in 995 individuals from 1KGP and also in an independent cohort of 308 Brazilian Euro-descendants. The association was only observed in individuals carrying at least one copy of HLA-C2, which suggests an orchestrated and refined evolution between these two systems. Although the antibody used in this study (DT9) cross reacts with HLA-E, it has been demonstrated that its binding represents the surface expression of HLA-C (28, 31) and also is correlated with mRNA expression levels of *HLA-C* measured by quantitative PCR (32). Therefore, our direct measurement of HLA-C expression in 30 individuals demonstrates that imputing HLA expression based on previously published data is predictive of the expression observed on the surface of fresh blood cells.

It is also interesting that the same allele rs2304224^*^T is associated with higher expression of the receptor KIR2DL1 in NK cells and also present in the high expressing KIR2DL1^*^002. The SNP *rs2304224* in exon 1 causes a non-synonymous substitution of valine (allele *G*) to phenylalanine (allele *T*) in the signal peptide. The hydrophobicity of the signal peptide can influence protein retention in the cytosol (33). According to the Wimley-White interfacial hydrophobicity scale (34), valine has a free energy of transfer of 0.07 ΔG from water to bilayer, and the free energy of phenylalanine is −1.13 ΔG. The lower and negative value of phenylalanine indicates this transference is more favorable, and therefore, rs2304224^*^T may increase protein availability in the membrane. This could explain the increased KIR2DL1 expression associated with rs2304224^*^T.

The patterns that we observed for the expression of KIR2DL1 allotypes (Supplementary Figure 4) are consistent with previous studies (20, 35–37). Our results showing that copy number of *KIR2DL1* affects the quantity of KIR2DL1^+^ NK cells corroborate those by Béziat et al. (37). On the other hand, the lack of significant association that we observed between *KIR2DL1* copy number and the abundance of expression on the cell surface reinforces the idea that copy number does not affect levels of KIR2DL1 as strongly as it affects the proportion of cells expressing the receptor (37). The presence of HLA-C2 was associated with lower expression of surface KIR2DL1, according to our results and of others (35, 38, 39). However, differently from the observations from Le Luduec et al. (38), who observed that the expression of KIR2DL1 is associated to the presence of C2 in a dose dependent manner, we found association only in individuals carrying two copies of *C2*.

We found three SNPs in LD with *rs2304224* (D′ = 0.99, 0.18≤ *r*^2^ ≤0.51). The low correlation coefficient is explained by difference in the allele frequencies among them. The frequency of the variant rs2304224^*^T is 0.26 in Euro-Brazilians, while the frequency of rs4806553^*^C is 0.67; rs687000^*^A is 0.57; and rs34721508^*^C is 0.86. From the three variants in LD with *rs2304224*, only *rs34721508*, in exon 7, has been previously associated with differential expression levels of KIR2DL1 in transfected cell lines (36). That study showed that cells expressing allotypes with 245^Cys^ have reduced protein stability and are more susceptible to ligand mediated expression down-regulation in comparison to those with 245^Arg^. Interestingly, this variant was also present in the 1KGP dataset, and we did not observe association of *rs34721508* genotypes with HLA-C imputed expression levels (*p* = 0.28). We also demonstrated that there is an additive effect of rs2304224^*^T and rs34721508^*^C on KIR2DL1 expression, which indicates that each has independent effect on the expression of KIR2DL1 (Supplementary Figure 6), despite the fact that both these variants are present in the high expressing KIR2DL1^*^002 (Supplementary Table 1). This observation argues in favor of our approach to expand the analysis of individual SNPs rather than solely analyzing the common combinations of SNPs present in the most frequent *KIR2DL1* alleles.

We applied extended haplotype homozygosity (EHH) analysis to all SNPs in LD with *rs2304224*, using the next generation sequencing data that we generated for a subset of Euro and Japanese descendants. Homozygosity surrounding the derived allele rs4806553^*^G was prominent in the Japanese population, suggesting this allele has been under recent positive selection. Japanese populations are especially interesting because they exhibit the lowest frequency of the HLA-C2 allotype (only 8%) (40) and, accordingly, we report low frequency of C2 also in the Brazilians of Japanese ancestry (10.3%). The low frequency of HLA-C2 could be driving the evolution of *KIR2DL1* in the Japanese population.

The SNP *rs4806553* is located 406 kbp upstream of the *KIR2DL1* gene, in the sequence corresponding to its intermediate promoter (Pro-I), suggested to control protein expression in mature NK cells (41). Moreover, it has been shown that the Pro-I sequence containing allele rs4806553^*^C binds to the transcription factor activator protein-1 (AP1), while rs4806553^*^G abrogates this binding (42). This could potentially explain the higher expression of KIR2DL1^*^002, which contains allele rs4806553^*^C, in comparison to other *KIR2DL1* alleles carrying the variant rs4806553^*^G, such as KIR2DL1^*^004 and KIR2DL1^*^006 (Supplementary Figure 4 and Supplementary Table 1). Our data suggests that the attenuation of NK inhibition mediated by KIR2DL1 represents an evolutionary advantage and is being favored by positive selection in the Japanese population.

Strong signals of positive selection were observed toward the derived allele rs687000^*^G in both our cohorts. This variant is a synonymous change in exon 4, without apparent impact on regulation of *KIR2DL1* expression. One hypothesis is that rs687000^*^G rose in frequency due to hitchhiking with a nearby variation that was positively selected and eventually fixed. We did not observe signals of positive selection for *rs2304224* and *rs34721508*, which strongly associate with KIR2DL1 expression levels. One possibility is that selection could be favoring specific *KIR2DL1* alleles that carry these variants. In fact, the combination of rs2304224^*^G (neutral), rs687000^*^G (positively selected), and rs34721508^*^C (neutral) defines KIR2DL1^*^003, the most frequent allele across all populations worldwide (43).

Coevolution of *KIR* and *HLA* is mostly driven by *HLA-C* (20, 44), which encodes a strong educator for KIR^+^ NK cells (45, 46). A fine tuning mechanism of NK cell regulation through the cell-specific promoter NK-Pro (47) was recently proposed, in which expression levels of HLA-C during NK cell education combines with expression levels and interaction strength of KIR and HLA in mature NK cells to modulate their selectivity and cytotoxicity (48). KIR2DL1 is the receptor with the highest affinity and avidity to HLA-C, and mediates the strongest NK response (19, 20, 49). Therefore, it is plausible that variation in *KIR2DL1* could be under selection and also that *KIR2DL1* and *HLA-C* are coevolving. Here, we show a *KIR2DL1* variant that is associated with lower expression of KIR2DL1 and inversely associated with higher HLA-C expression in HLA-C2/C2 individuals. This could be an indication that higher levels of the ligand are being compensated by lower expression of the receptor. We also observed evidence of positive selection on KIR2DL1. Our data show that much remains to be understood regarding the mechanisms of the KIR-HLA recognition and evolution. They also bring insights into the evolution of these two systems and suggest that more questions will emerge as we explore more deeply *KIR-HLA* diversity at high resolution.

## Materials and Methods

### Samples

We analyzed a cohort of 308 individuals of predominantly European ancestry and 75 individuals of Japanese ancestry from Curitiba, Brazil. About 80% of the population from Curitiba self-reported as Euro-descendant (50), which is in accordance with previous genetic studies (51). For the Japanese descendants, we only included individuals who had two parents or four grandparents born in Japan, with no history of admixture with non-Japanese ancestries. In order to measure KIR2DL1 expression levels, we analyzed fresh blood cells from a subset of 48 Euro-descendants. A subset of 30 of these individuals were included in the HLA-C expression assay. Detailed information about the study design is given in Supplementary Figure 7. All individuals were living in Curitiba, Brazil, at the time of blood collection. Median age in the group was 26 years (ranging from 20 to 64) and the male/female ratio was 0.37.

For expression assays, we collected 8 mL of peripheral blood samples and isolated PBMC (peripheral blood mononuclear cells) using Leucosep^TM^ tubes (Greiner Bio-One, Austria), which have a selective membrane for density-based lymphocyte separation, and Ficoll Hypaque (Sigma Aldrich, MO). Isolated PBMC were counted in a Neubauer chamber under an optical microscope. A total of 0.5 × 10^6^ cells were incubated with specific antibodies for KIR2DL1 and HLA-C and analyzed by flow cytometry. Detailed description and gate strategy are shown in Supplementary Figure 8.

### *KIR2DL1* and *HLA-C* Genotyping

We initially sequenced exons 1, 4, 5, 7, and 9 to distinguish the main *KIR2DL1* allele groups using the Sanger method (52) in the 48 Euro-descendants included in the expression assay (Supplementary Figure 9). The sequences obtained were aligned with reference sequences from IPD-KIR database (43), using the software Mutation Surveyor® (SoftGenetics, PA) and identified manually. Additionally, we sequenced only the exon 1 (containing the variant *rs2304224*) in extra 260 Euro-descendant individuals to increase statistical power for the analysis of *rs2304224*.

We applied quantitative PCR to determine copy number of *KIR2DL1* compared to *KIR3DL3*, which is present in virtually all haplotypes. *KIR2DL1* was amplified in triplicate using one set of primers and the reference gene *KIR3DL3* was amplified using other three sets of primers, each in triplicate, in a total of 12 (4 × 3) reactions per sample. The sequence of all primers used for amplification, sequencing and copy number assay, including those designed in this study as well as those described previously (53–57) are given in Supplementary Table 2.

We also sequenced the entire *KIR2DL1* gene in 109 Euro-descendants and 75 Japanese descendants from Curitiba, Brazil. These samples were sequenced using the previously published method for next generation sequencing of *KIR* and *HLA* genes (29) using Illumina platform.

### Data Analysis

Normality of variables was tested using Kolmogorov-Smirnov test, in R package *nortest* (58). Difference in HLA-C expression between *KIR2DL1* SNP genotypes was tested via the Kruskal-Wallis test, using *stats* R (59). *Post-hoc* analysis of Dunn was applied to Kruskal-Wallis results in order to identify pair-wise significant differences between genotypes, in R package *dunn.test* (60). Median HLA-C expression by allele, as defined by Apps et al. (28), was imputed for each allele in an individual, and then summed. The imputation was performed in all 308 Brazilians of European ancestry sequenced for *rs2304224* and 1KGP individuals. Correlation analysis between expected HLA-C expression in CD3^+^ cells and *in vivo* HLA-C expression in CD3^+^ cells was calculated with R package *Hmisc* (61). Difference in KIR2DL1 expression according to copy number was tested using Mann-Whitney, in *stats* R (59). Association of KIR2DL1 expression with allotype and *rs2304224* was tested through logistic regression using copy number as a covariate, also in *stats* R. Linkage disequilibrium was estimated using LD function from R package *genetics* (62) and plotted with a modified version of R package *LDheatmap* (63). Median expression graphs were plotted using *base* and *beeswarm* R packages (59, 64).

*KIR2DL1* SNPs obtained from genomic sequence data were phased using fastPHASE, with modified parameters (-T10 -H200). The phased data was used for estimation of extended haplotype homozygosity (EHH) (65) using R package *rehh* (66). Ancestral and derived alleles were defined according to the Database of Single Nucleotide Polymorphisms (dbSNP) (67).

## Data Availability Statement

All datasets generated for this study are included in the article/Supplementary Material.

## Ethics Statement

The studies involving human participants were reviewed and approved by Brazilian National Human Research Ethics Committee (CONEP), Protocol No. CAAE 02727412.4.0000.0096, in accordance to the Brazilian Federal laws. The patients/participants provided their written informed consent to participate in this study.

## Author Contributions

DA designed the study. LV, RD, VC-S, LA, and HI performed Sanger sequencing and genotyping. LV, DA, and BH performed next generation sequencing. LV, RD, and DA performed flow cytometry analysis. LV, DA, and WM analyzed the data. MP-E, JH, and DA contributed with samples and/or reagents. LV and DA drafted the manuscript. All authors contributed to the article and approved the submitted version.

## Conflict of Interest

The authors declare that the research was conducted in the absence of any commercial or financial relationships that could be construed as a potential conflict of interest.
