# Supplementary material for: Single Nucleotide Polymorphism in KIR2DL1 Is Associated With HLA-C Expression in Global Populations
Source: Front Immunol. 2020 Aug 21;11:1881. doi: 10.3389/fimmu.2020.01881 (PMC7478174; doi:10.3389/fimmu.2020.01881)
Supplement: Supplementary file 1 [file Data_Sheet_1.pdf]

Supplementary Material

1 Supplementary Figures and Tables

1.1 Supplementary Figures

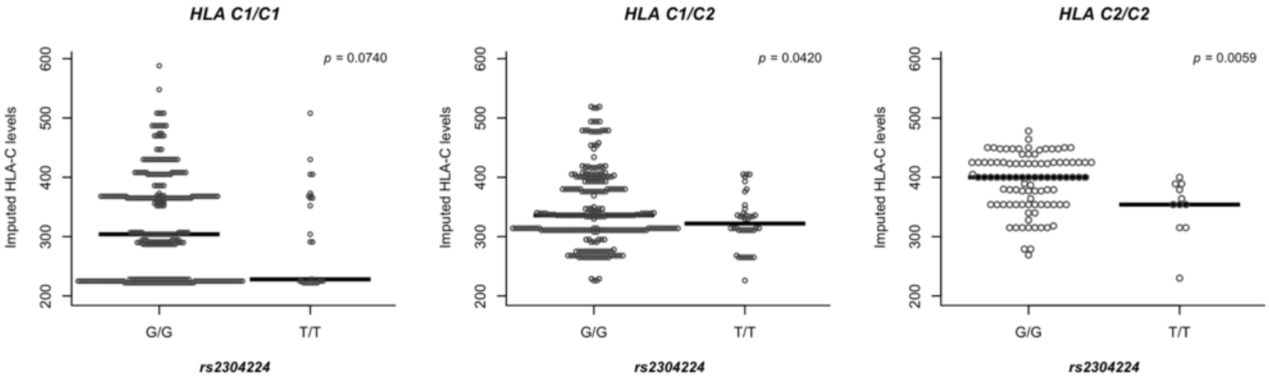

Supplementary Figure 1. Associations of *rs2304224* and HLA-C expression levels in 1KGP samples. (A) Non-significant differences between *G/G* and *T/T* genotypes of *rs2304224* were observed in donors carrying two copies of HLA-C1 (p=0.0740). (B) Allele *rs2304224*T* is associated to HLA-C expression in both heterozygous C1/C2 (p=0.0420) and (C) homozygous C2/C2 (p=0.0059). Each dot in the graphs represents one individual. Median values are shown in horizontal lines and p-values in the top right corner. HLA data were previously published (27) for the samples of the 1000 Genomes Project (1KGP). Median HLA-C expression by allele, as defined by Apps et al. (28), was imputed for each individual genotype.

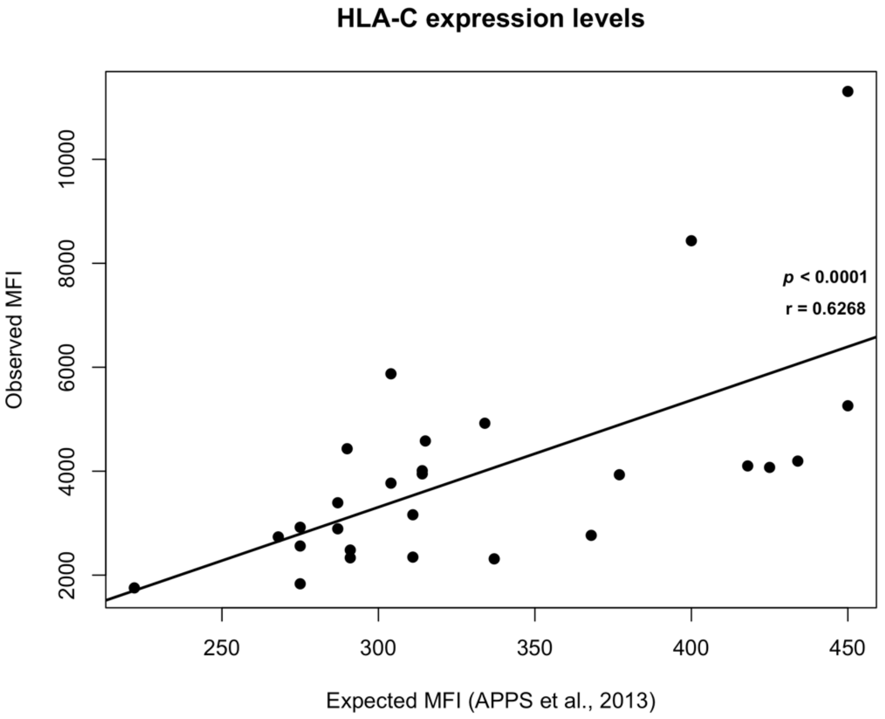

Supplementary Figure 2. Correlation fit between HLA-C expression in fresh CD3+ cells compared to imputed HLA-C expression levels also from CD3+ cells, as described in Apps et al. (28)**.** MFI: median fluorescence intensity.

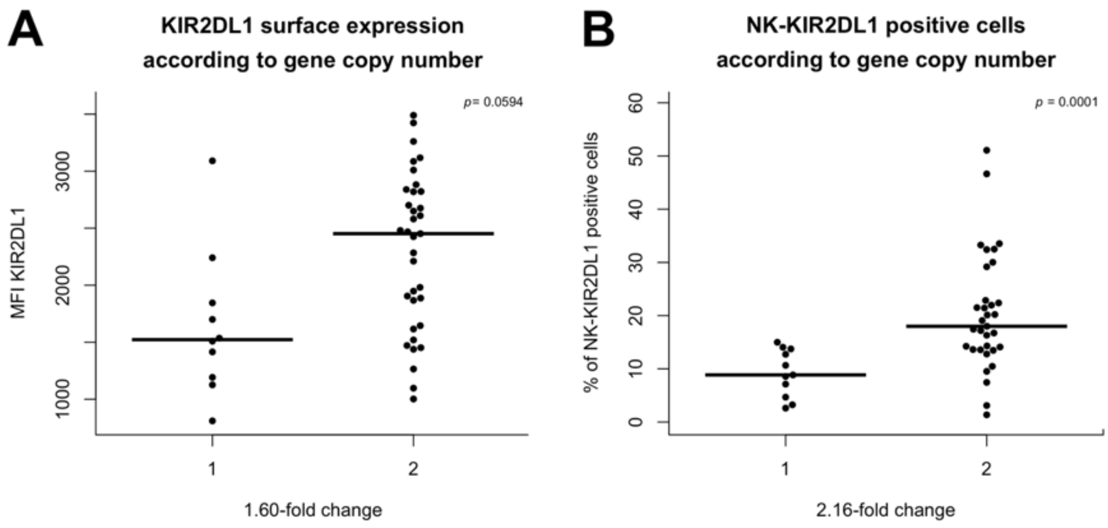

Supplementary Figure 3. The effect of gene copy number on KIR2DL1 expression. *KIR2DL1* copy number was not associated with (A) abundance of KIR2DL1 on the surface of NK cells (*p*=0.0594) but was associated with (B) percentage of NK cells positive for KIR2DL1(*p*=0.0001). Gene copy number is indicated in the x axis: 1 copy (hemizygosity) or 2 copies (homozygosity or heterozygosity). Each dot in the graphs represents one individual. Median values are shown in horizontal lines and *p*-values in the top right corner.

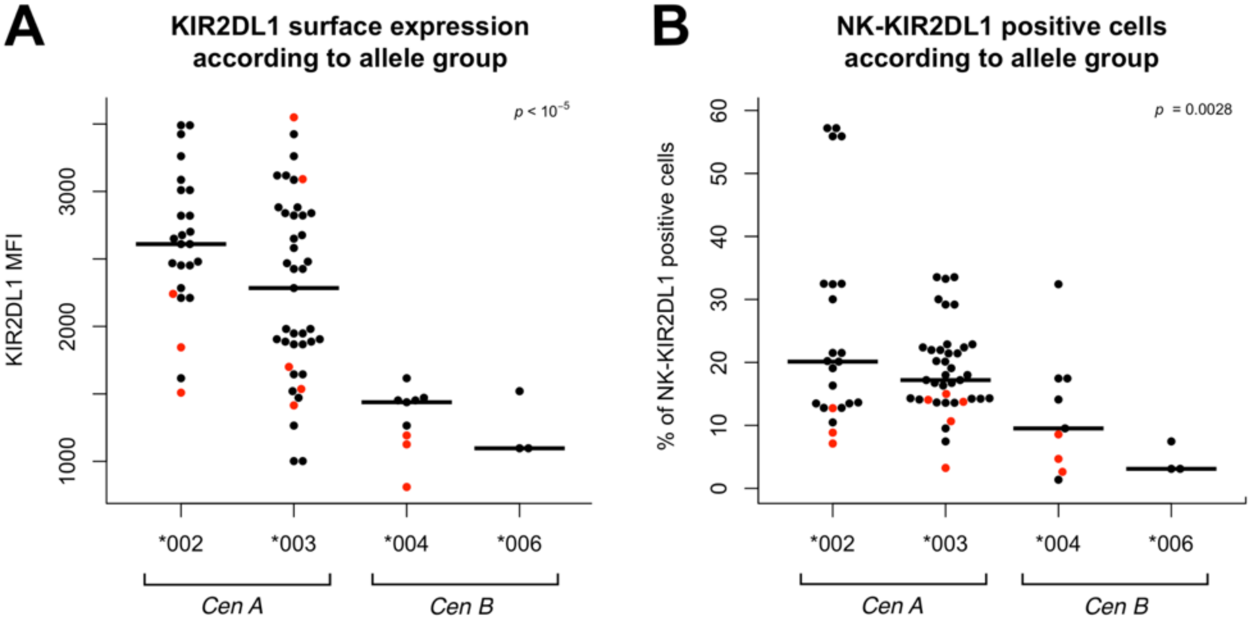

Supplementary Figure 4. Differential expression of common KIR2DL1 allotypes. *KIR2DL1* allelic diversity is associated with (A) abundance of KIR2DL1 on the surface of NK cells ($p < 10^{-5}$) and to (B) percentage of NK cells positive for KIR2DL1 ($p = 0.0028$). Each dot in the graphs represents one individual, which is plotted twice (once for each allele). Red dots indicate hemizygosity for *KIR2DL1*. Median values are shown in horizontal line. *p*-values in the top right corner of each graph indicate the significance of differences in expression among all allele groups given by the Kruskal-Wallis test. Post-hoc Dunn test shows that there is no significant difference within *Cen A* alleles *(*002* and **003*) or within *Cen B* alleles (**004* and **006*) (p > 0.05), but there is a difference between *Cen A* and *Cen B* alleles in all pairwise comparisons ($p < 10^{-2}$).

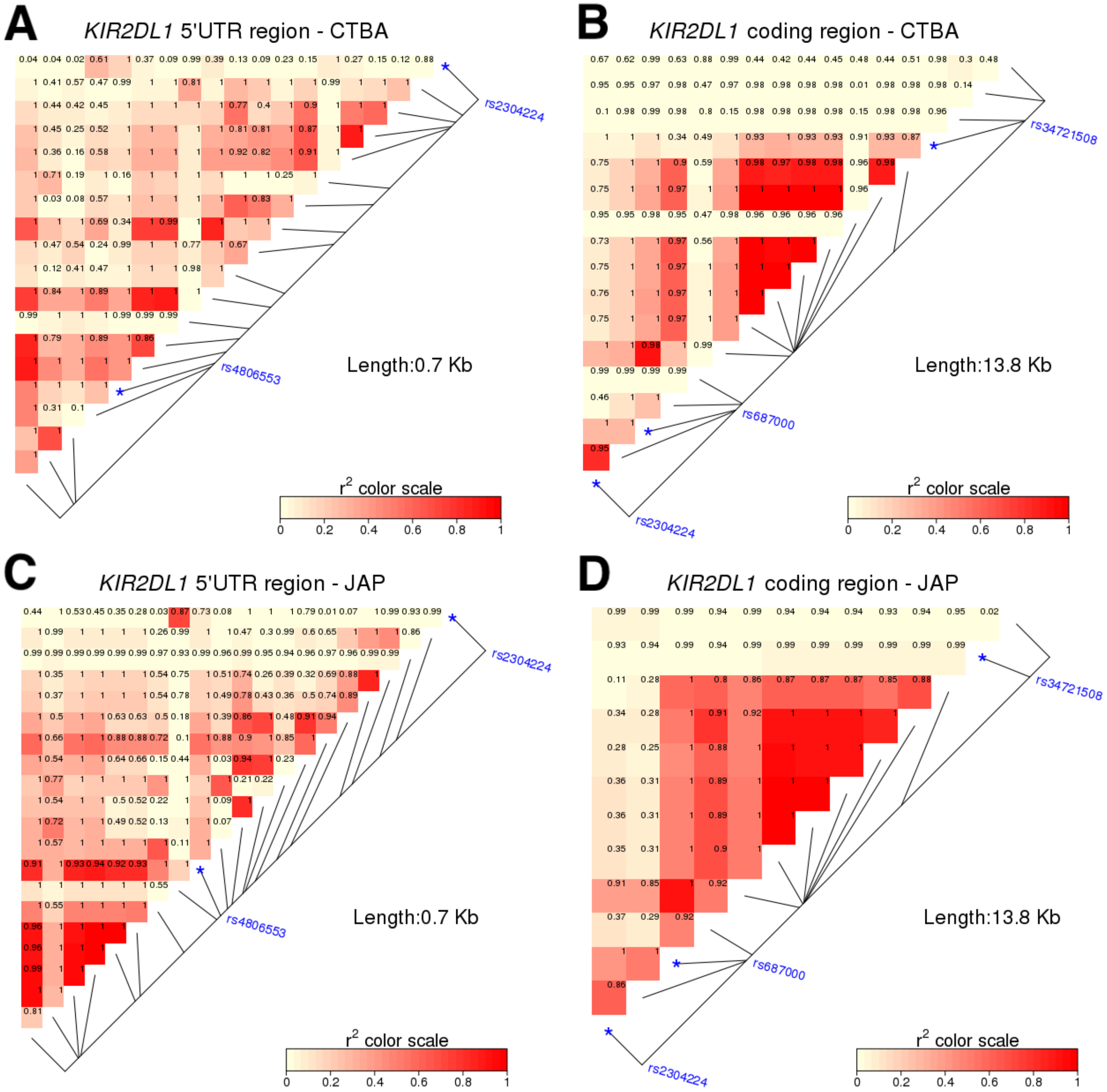

Supplementary Figure 5. Linkage disequilibrium (LD) between *KIR2DL1* polymorphisms. (A) LD spanning 700 bp in the 5' untranslated region (UTR) up to *rs2304224*, and (B) LD across *KIR2DL1* coding region in Euro-Brazilians (n=109). (C) LD spanning the 5' untranslated region (UTR) and *rs2304224*, and (D) LD across *KIR2DL1* coding region in Japanese (n=75). Cell color corresponds to the correlation coefficient (r^2) between markers, and normalized LD coefficient (D') is given by the number inside each cell. Asterisks and their respective *rs*ID accession numbers highlight all SNPs in significant linkage disequilibrium with *rs2304224* (p<0.01).

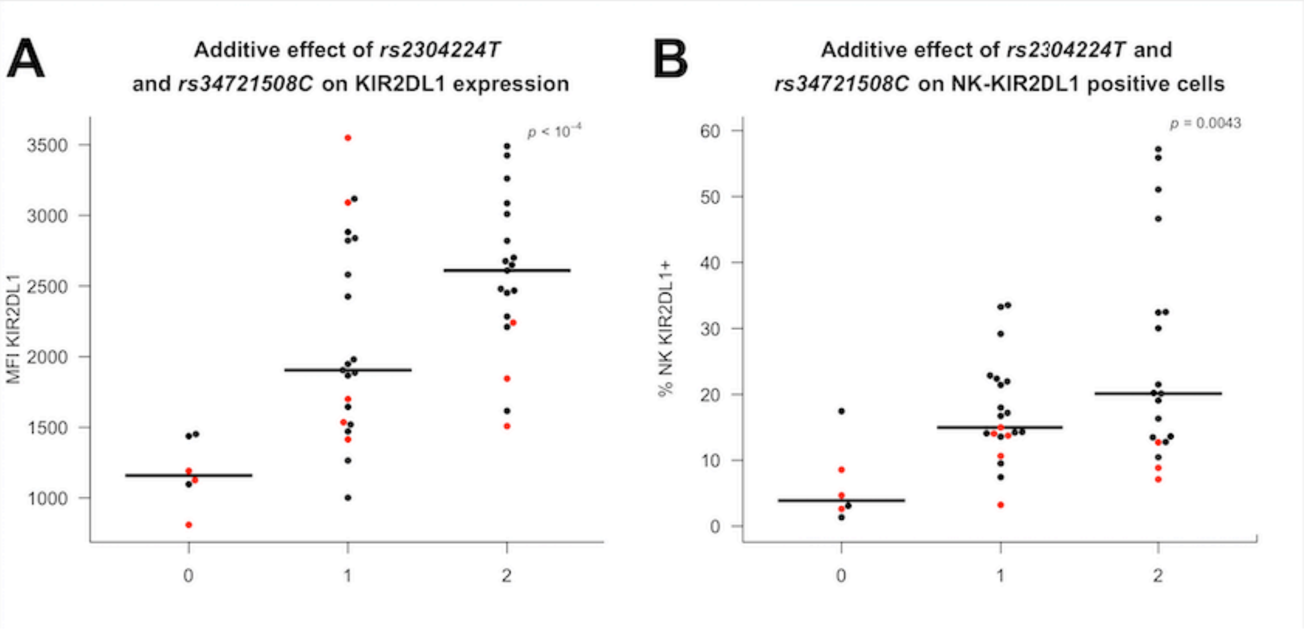

Supplementary Figure 6. Additive effect of *rs2304224*T* and *rs34721508*C* on KIR2DL1 expression. (A) KIR2DL1 surface expression ($p < 10^{-4}$) and (B) the percentage of NK cells KIR2DL1 positive ($p = 0.0043$). Each dot in the graphs represents one individual, which is plotted twice (once for each allele). Red dots indicate hemizygosity for *KIR2DL1*. The score of 2 in the *x* axis represents individuals who have both *rs2304224*T* and *rs34721508*C*; while 1 indicates those who have only *rs2304224*T* or *rs34721508*C*; and the score 0 is given when both variants are not present. Median values are shown in horizontal line. *p*-values in the top right corner of each graph indicate the significance of differences in expression among all allele groups given by the Kruskal-Wallis test.

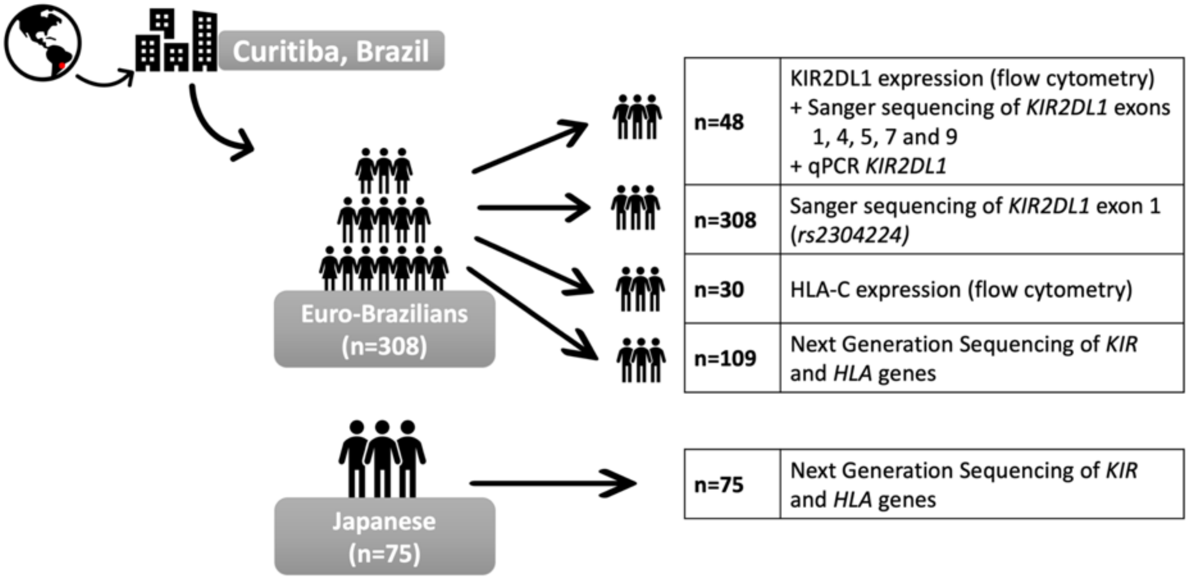

Supplementary Figure 7. Study design. All samples were collected in Curitiba, in the south of Brazil.

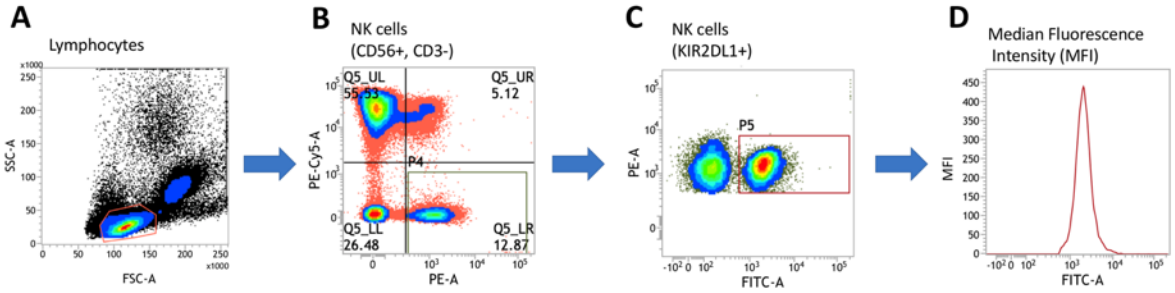

Supplementary Figure 8. Gating strategy for flow cytometry analysis. For KIR2DL1 expression, a total of 0.5×10^6 cells were incubated with the following antibodies: anti-CD56 (REA196 clone PE conjugated, Miltenyi Biotec Cat# 130-100-622, RRID:AB_2658731), anti-CD3 (HIT3a clone PE-Cy5 conjugated, Biolegend Cat# 300328, RRID:AB_1575008) and the specific anti-KIR2DL1 antibody (REA clone FITC conjugated, Miltenyi Biotec Cat# 130-103-967, RRID:AB_2655323). Cells were washed with PBS 1X solution after incubation and then run through BD FacsVerse^{TM} cytometer. The cytometer was calibrated and compensated using CS&T beads (BD Biosciences Cat# 650621). Gating and cell populations were defined using the equipment's software BD FacSuite^{TM} (BD Biosciences, CA). (A) First, lymphocytes were selected from the PBMC pool based on size (FSC-A) and granularity (SSC-A). Each dot represents one cell, as the density of cells occupying the same position in the graph increases, the color changes from blue to red. (B) Then, we selected lymphocytes which were CD56 positive (PE-A) and CD3 negative (Pe-Cy5-A), corresponding to NK cells. (C) From the NK cell pool, cells positive for KIR2DL1 (FITC-A) were selected. The count of positive and negative cells in this stage determine the percentage of cells expressing KIR2DL1. (D) The expression of KIR2DL1 is given using the median fluorescence intensity (MFI) for each individual. A number of 1,000 target nuclei events were acquired per sample, given the rare occurrence of NK-KIR2DL1$^+$ cells in PBMC (lymphocytes are present in ~80% of PBMC, ~8.7% of those are NK cells, and a median of 15.65% of NK cells are positive for KIR2DL1, which makes a total of 1.09% NK-KIR2DL1$^+$ cells in the PBMC pool). A similar approach was used to measure HLA-C expression levels in a subset of 30 individuals using anti-HLA-C antibody (DT9 clone with no fluorescence, BD Biosciences Cat# 566372, RRID: AB_2739715), with the secondary antibody anti-mouse IgG2ab (X-57 clone FITC conjugated, Miltenyi Biotec Cat# 130-098-126, RRID:AB_2661521), and a third antibody anti-CD3 (HIT3a Clone PE/Cy5 conjugated, Biolegend Cat# 300309, RRID:AB_314045). The acquisition was modified to 10,000 target data events acquired per sample, given the high abundancy of cells containing the HLA-C receptor in the CD3$^+$ pool.

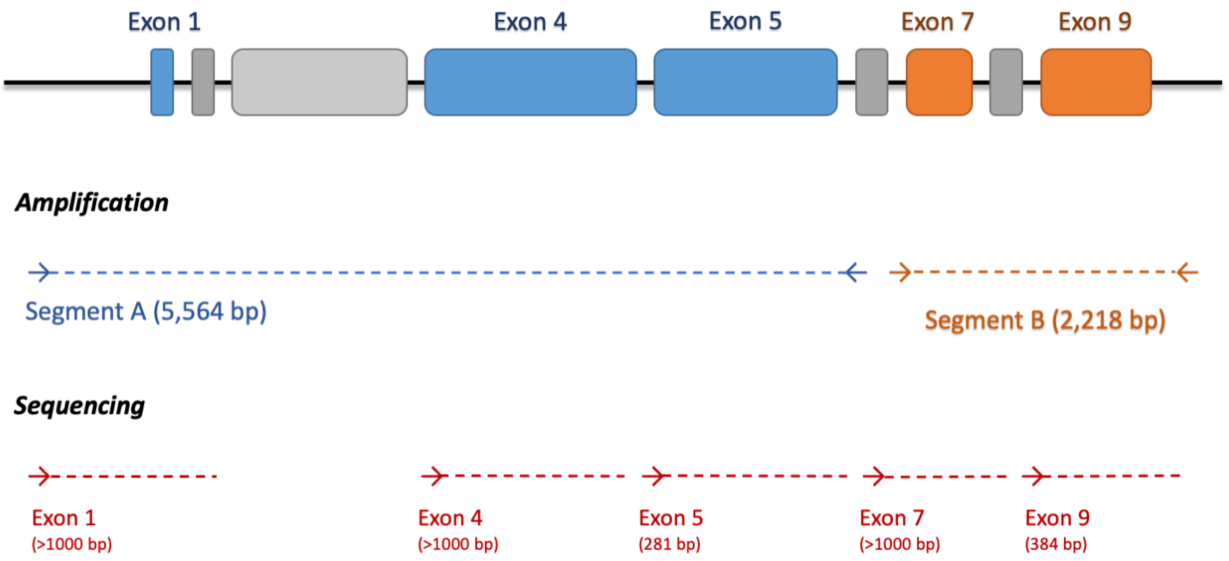

Supplementary Figure 9. Strategy used to sequence *KIR2DL1* using Sanger. We sequenced exons 1, 4, 5, 7 and 9, which distinguish between the main *KIR2DL1* allele groups. Two long amplification reactions were performed. The first segment comprised exons 1 to 6 (5,564 bp) and was amplified using GoTaq® Long PCR Master Mix (Promega, WI) with 1 cycle at 94˚C for 3 minutes, 35 cycles (94˚C for 20 seconds, 59˚C for 30 seconds, 68˚C for 5 minutes) and 1 cycle at 68˚C for 10 minutes. The second amplification included exons 7 to 9 (2,218 bp) and it was amplified using Taq Platinum (Invitrogen, CA). The reaction conditions were 1 cycle at 95˚C for 5 minutes, 35 cycles (95˚C for 20 seconds, 61˚C for 30 seconds and 68˚C for 5 minutes) and 1 cycle at 68˚C for 5 minutes. The amplification products were purified with Exonuclease I (Fermentas, MA) and alkaline phosphatase (Thermo Fisher Scientific, MA) and subsequently used for the five individual sequencing reactions of exons using master mix Big Dye Terminator Cycle Sequencing Standard v3.1 (Life Technologies), for 1 cycle at 95˚C for 10 seconds and 35 cycles (50˚C for 5 seconds and 60˚C for 4 minutes) and 1 cycle at 68˚C for 10 minutes. The sequencing product was purified by precipitation and resuspended in 10 μL of Hi-Di Formamide (Life Technologies, CA). Capillary electrophoresis was performed using 3500xl Genetic Analyzer (Life Technologies, CA).

1.2 Supplementary Tables

	rs4806553		***rs2304224***		*rs687000*		*rs34721508*	
CDS position	-406		**13**		144		796	
Protein codon	non-coding		-17 (sig. peptide)		27		245	
Protein substitution	non-coding		V>F		synonymous		R>C	
Allele	*C**	*G*	*G**	*T*	*A**	*G*	*C**	*T*
Euro-descendants	0.67	0.33	0.74	0.26	0.57	0.43	0.86	0.14
Japanese	0.44	0.56	0.89	0.11	0.23	0.77	0.99	0.01
	rs4806553		***rs2304224***		*rs687000*		*rs34721508*	
High expressing allotypes								
KIR2DL1*002	C		T		A		C	
KIR2DL1*003	G or C		G		G		C	
Low expressing allotypes								
KIR2DL1*004	G		G		A		T	
KIR2DL1*006	G		G		A		T	

Supplementary Table 1. *KIR2DL1* variants and frequencies. Allele frequencies of *rs2304224* and linked SNPs in Euro-Brazilians (n=109) and Japanese (n=75) populations, as well as their corresponding alleles in common KIR2DL1 allotypes. CDS: coding sequence (DNA). Asterisks indicate the ancestral allele of each SNP. Frequencies corresponding to the alleles which appear to be under positive selection (*rs4806553*G* and *rs687000*G*) are indicated in red.

Reaction	Gene	Pair	Sequence (5' – 3')	Reference (if applicable)
Pre-sequencing amplification	*KIR2DL1*	A	**GGACACTAGGTGTCAAATTCTAGC**	
			**ACAAGCAGTGGGTCACTTGAC**	
		B	**TCACAGGAGGACAGGTGGTT**	
			**GCTGTTGTCTCCCTAGAAGACG**	
Sequencing	*KIR2DL1*	Exon 1	**GGACACTAGGTGTCAAATTCTAGC**	
		Exon 4	**GAAGGAGAGAGATAAGACACCAGG**	
		Exon 5	**GAAGATCCTCCCTGAGGAAAC**	
		Exon 7	GGGTGCTTGTCCTAAAGAGG	(54)
		Exon 9	**GGACCCAGAAGTGCCCTC**	
Presence/absence typing	*KIR2DL1*	17B	CCATCAGTCGCATGACG	(55)
			TCACTGGGAGCTGACAC	
		16	TGGACCAAGAGTCTGCAGGA	(56)
			TGTTGTCTCCCTAGAAGACG	
	HLA-DRB1	Control	TGCCAAGTGGAGCACCCAA	(57)
			GCATCTTGCTCTGTGCAGAT	
Copy number	*KIR2DL1*	17B	CCATCAGTCGCATGACG	(55)
			TCACTGGGAGCTGACAC	
		ddPCR	GTTGGTCAGATGTCATGTTTGAA	(58)
			CCTGCCAGGTCTTGCG	
			**AACGACACTTTGCGCCTCATT**	
	KIR3DL3	1C	TTCTGCACAGAGAGGGGATCA	(56)
			GAGCCGACAACTCATAGGGTA	
		2B	CACTGTGGTGTCTGAAGGAC	(55)
			GGAGTGTGGGTGTGAACTG	
		RT	CTGCAATGTTGGTCAGATGTCAG	Modified from (58)
			GGGAGCYGACAACTCATAGGGTA	
			GCTCTGGTTGTAGTAGCCGCGCAG+T	

Supplementary Table 2. Primers used in *KIR2DL1* analysis. Sequences in bold were designed in this study.
